# Organ Crosstalk During Injury: Mechanisms of Lung–Kidney Interaction in Critical Illness

**DOI:** 10.1002/cph4.70069

**Published:** 2025-12-03

**Authors:** Kathryn M. Sullivan, Kathleen D. Liu, Michael A. Matthay

**Affiliations:** ^1^ Department of Medicine University of California San Francisco San Francisco California USA; ^2^ Department of Anesthesia and Perioperative Care University of California San Francisco San Francisco California USA; ^3^ Cardiovascular Research Institute University of California San Francisco San Francisco California USA

**Keywords:** acute kidney injury (AKI), acute lung injury, acute respiratory distress syndrome (ARDS), critical illness, organ crosstalk

## Abstract

The kidneys and lungs are frequent sites of organ injury during critical illness. Acute kidney injury (AKI) and acute respiratory distress syndrome (ARDS) are clinical syndromes resulting from kidney and lung injury respectively. Complex pathophysiologic mechanisms underlie the development of these two syndromes individually, and a substantial body of evidence now indicates that crosstalk between the lungs and the kidneys occurs after organ injury. Here we review the pathophysiology of AKI and ARDS, animal models of kidney and lung injury, and mechanisms of organ crosstalk after injury has occurred. We focus the discussion on how either kidney injury or lung injury may propagate damage in the other organ, which is relevant to multiorgan injury commonly encountered in the intensive care unit. The reviewed literature contains more mechanistic preclinical studies of lung injury after AKI compared with AKI after lung injury. Identified mechanisms of lung injury after AKI include leukocyte recruitment, inflammatory signaling, activation of pattern recognition receptors, formation of neutrophil extracellular traps, osteopontin signaling, metabolic dysfunction, and impaired alveolar fluid clearance. After lung injury, AKI is instigated by inflammatory signaling, the effects of mechanical ventilation, and consequences of fluid management.

## Graphical abstract

Crosstalk between the lungs and kidneys may propagate multiorgan injury in critically ill patients. This review examines evidence of crosstalk between the lungs and kidneys after injury, focusing on translational and mechanistic studies. Pathophysiology and animal models of acute kidney injury (AKI) and acute respiratory distress syndrome (ARDS) are discussed.

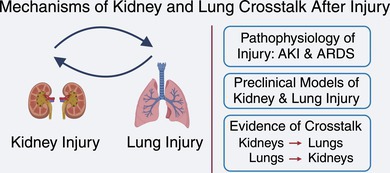

## 1 Introduction

Under the physiologic and clinical stress of critical illness, interorgan communication is a key aspect of both pathology and recovery. Pulmonary and renal function are particularly intertwined. The lungs and kidneys are common sites of organ injury in critical illness and concurrent injury is frequent. Acute respiratory distress syndrome (ARDS) represents the most advanced form of lung injury in which an underlying inflammatory insult causes rapid development of increased permeability, generating pulmonary edema that is not cardiogenic in nature (Wick et al. 2024). Edema fluid in the alveolar spaces leads to impaired gas exchange with severe hypoxemia and reduced carbon dioxide excretion, often requiring mechanical ventilation. Acute kidney injury (AKI) occurs across a spectrum of severity and can lead to fluid retention, acidosis and impaired clearance of electrolytes and metabolic byproducts (Ostermann et al. 2025). In the extreme, AKI can require initiation of renal replacement therapy (RRT). A growing body of evidence has established that crosstalk between the lungs and the kidney is important to the development of organ injury (Faubel and Edelstein 2016; Komaru et al. 2024; Joannidis et al. 2020; Matsuura et al. 2023; Kumar et al. 2024). Here we review mechanisms by which injury to either the lungs or kidneys affects the function of the other organ with a focus on critical illness.

Though crosstalk occurs across many organ systems, interactions between the lungs and kidneys are compelling to study from a public health perspective because these organs are frequent sites of injury for critically ill patients and they are often injured simultaneously. Independently, ARDS and AKI are highly morbid conditions, and patient outcomes are worse when they occur together (McNicholas et al. 2019; Liu et al. 2007; Darmon et al. 2014; Federspiel et al. 2018; Chawla et al. 2017; Tomasi et al. 2023). The incidence of AKI is higher among ICU patients with ARDS compared with those without ARDS (Darmon et al. 2014). Approximately 39%–44% of patients with ARDS but without pre‐existing renal disease develop AKI during hospitalization (McNicholas et al. 2019; Darmon et al. 2014). ARDS patients who develop AKI have a significantly greater mortality compared with ARDS patients without AKI (McNicholas et al. 2019; Liu et al. 2007; Darmon et al. 2014; Federspiel et al. 2018). AKI itself may contribute to pulmonary injury and ARDS risk (Faubel and Edelstein 2016). In predictive modeling of patients with severe AKI, respiratory complications are associated with a substantial increase in mortality compared with other clinical factors affecting outcomes (Demirjian et al. 2011).

Fundamentally, ARDS and AKI are both heterogeneous clinical syndromes that result from a variety of acute, pathophysiologic triggers that are overlapping (Wick et al. 2024; Ostermann et al. 2025). Despite clear evidence that these clinical disorders are interrelated, interorgan communication can be difficult to isolate in the presence of shared underlying risk factors for organ injury (Joannidis et al. 2020; Borges and Bento 2024). For instance, in the case of a patient with septic shock and ARDS due to pneumococcal pneumonia, to what extent does pulmonary tissue injury itself contribute to the risk for AKI development versus the underlying pathophysiology of septic shock? Conversely, in a patient with AKI in the setting of hemorrhagic shock from trauma, to what extent does ischemic injury of renal tissue contribute to the risk for ARDS versus the underlying trauma or treatment with transfusion therapy? Separating out the timing of various organ injuries in relation to one another is a limitation to fully understanding crosstalk through clinical investigation.

Despite these challenges, through a combination of carefully designed clinical studies and experimental animal models of organ injury, substantial insight has grown regarding specific mechanisms of interorgan interaction in the setting of critical organ injury. Kidney injury impacts lung pathophysiology through a wide range of mechanisms including leukocyte recruitment, inflammatory cytokines, pattern recognition receptors (PRRs), neutrophil extracellular traps (NETS), osteopontin, mitochondrial dysfunction, and altered fluid transport. Lung injury impacts kidney pathophysiology through inflammatory cytokines, mechanical ventilation, and fluid management.

## 2 Definitions of Injury: Clinical Features and Pathogenic Mechanisms

### 2.1 Acute Respiratory Distress Syndrome and Acute Lung Injury

#### 2.1.1 Clinical Characteristics

ARDS is characterized by bilateral pulmonary edema resulting in severe hypoxia that occurs within 1 week of a clinical insult and is not fully explained by cardiac failure or volume overload. ARDS diagnostic criteria were redefined in 2024 with the release of the Global Definition of ARDS (Wick et al. 2024) which was an update of the prior Berlin Definition from 2012 (Ranieri et al. 2012). Under the Berlin ARDS definition, the oxygenation criteria required a PaO_2_/FiO_2_ ≤ 300 mmHg (ratio of arterial partial pressure of oxygen to fraction of inhaled oxygen) measured on a minimum PEEP of 5 cm H_2_0. The Global Definition expanded on the Berlin Definition to formally include patients on high flow nasal oxygen, and the oxygenation criteria can now be met using peripheral oxygen saturation when arterial blood gas sampling is not possible (SpO_2_/FiO_2_ ≤ 315). The term Acute Lung Injury (ALI) was used commonly in the literature prior to the implementation of the Berlin Definition of ARDS. Under the 1994 American European Consensus Conference (AECC) ARDS Definition, ALI was reserved for patients who had the features of ARDS but with milder hypoxemia (i.e., PaO_2_/FiO_2_ ratio of ≥ 200 mmHg). Patients who would have had ALI by the AECC Definition are considered to have mild ARDS under the Berlin and Global Definitions. For the purposes of this review, we refer to ARDS models in animals with the term lung injury which is distinct from this previous clinical definition of ALI. Management of ARDS is centered on treating the underlying clinical insult and supportive care using evidence‐based ventilatory management and fluid strategies, as no pharmacotherapies for ARDS currently exist (Wick et al. 2024).

#### 2.1.2 Pathogenesis

ARDS is an inflammatory process that develops after an acute clinical insult including sepsis (pulmonary and non‐pulmonary infection), shock, aspiration, trauma, transfusion and pancreatitis (Huppert et al. 2019). The risk for developing ARDS in the setting of one of these acute processes is impacted by environmental risk factors including air pollution, tobacco smoking and alcohol abuse (Wick et al. 2024). Chronic medical comorbidities including heart failure, severe liver disease, immunocompromise, malignancy, and chronic neurologic disorders impact ARDS outcomes (Wick et al. 2024; Rezoagli et al. 2022). Central to ARDS pathogenesis is alveolar epithelial and capillary endothelial injury which increases permeability and allows accumulation of protein‐rich edema fluid in the alveolar spaces, impairing gas exchange (Huppert et al. 2019). A variety of processes contribute to the pathogenesis: activation of the innate immune system, cytokine release, disruption of cell–cell junctions, leukocyte migration and impaired ion transport for the resolution of alveolar edema (Huppert et al. 2019).

### 2.2 Acute Kidney Injury

#### 2.2.1 Clinical Characteristics

AKI is defined by the 2012 Kidney Disease: Improving Global Outcomes (KDIGO) guidelines which are utilized across clinical practice and research applications (Khwaja 2012). AKI is indicated by at least one of the following criteria: an increase in serum creatinine by ≥ 0.3 mg/dL in 48 h, an increase in serum creatinine ≥ 1.5 times baseline within 7 days, or oliguria with urine volume ≤ 0.5 cc/kg/h for 6 h. AKI occurs in several acute clinical scenarios that are frequent in critically ill patients including sepsis, heart failure, liver disease, shock, following major surgery or due to nephrotoxic medications (Ostermann et al. 2025; Juncos et al. 2022). As in ARDS, supportive care is the keystone of management for AKI since there are no specific pharmacotherapies for AKI apart from vasoconstrictive therapy for hepatorenal syndrome (Pitre et al. 2022). The focus of treatment is addressing the underlying precipitant, preventing additional renal injury, correcting fluid and electrolyte imbalances and mechanical organ support via renal replacement therapy if necessary (Ostermann et al. 2025). Notably the KDIGO definition of AKI is functional and not based on diagnostic evidence of structural damage to the kidneys.

#### 2.2.2 Pathogenesis

At baseline, the kidney requires high metabolic activity to maintain solute transport and the concentration gradient for water reabsorption. In normal physiology, renal blood flow is directed to the glomerular circulation which is concentrated in the renal cortex (Zafrani et al. 2015; Post et al. 2017). Efferent arterioles leaving the juxtamedullary glomeruli branch off to form the vasa recta capillaries which then supply blood to the renal medulla (Zafrani et al. 2015; Post et al. 2017). This anatomy creates a scenario where there is decreasing oxygen tension and relative hypoxia in the medulla compared with the cortex (Zafrani et al. 2015). Due to this combination of anatomy and high oxygen demands under stress, portions of the kidney are particularly prone to hypoxemic insults.

AKI develops due to numerous pathophysiologic mechanisms that can overlap depending on the underlying contributors to injury and preexisting risk factors (Ostermann and Liu 2017). Broadly, these mechanisms can be grouped into ischemic (macrovascular, microvascular, congestion), inflammatory, and nephrotoxic (Juncos et al. 2022). Macrovascular dysfunction, that is, overt renal hypoperfusion from hemodynamic instability or surgical complications, is an established risk factor for AKI that is often clinically apparent. Microcirculatory dysfunction, however, is increasingly understood as a key feature of AKI, particularly in inflammatory diseases like sepsis (Zafrani et al. 2015; Post et al. 2017). Localized regions of ischemia can occur from severe microcirculatory disruption, even if global renal perfusion is maintained (Zafrani et al. 2015; Post et al. 2017). Microvascular thrombi contribute to microcirculatory dysfunction under inflammatory conditions that damage the endothelial barrier. Renal venous congestion impairs flow across the glomerulus and contributes to AKI, particularly in heart failure. Inflammatory injury and leukocyte recruitment can propagate AKI either due to a primary inflammatory insult like sepsis or in response to cell damage from other causes. Renal tubular cells are prone to injury through ischemia and inflammation, but are also at risk for direct injury via the tubular filtrate that may contain nephrotoxic medications or toxic cellular by‐products. Other mechanisms that may contribute to AKI in critically ill patients but are less commonly encountered include: tubular obstruction from crystal nephropathy or urinary tract obstruction; autoimmune conditions that lead to immune complex deposition; glomerulonephritis or vasculitis; abdominal compartment syndrome generating extreme venous congestion; or hypersensitivity to medications leading to acute interstitial nephritis (Ostermann and Liu 2017).

### 2.3 Sepsis

Sepsis is worth a special discussion in the context of organ crosstalk given that sepsis is the most common risk factor for both ARDS and AKI (Uchino et al. 2005; Hoste et al. 2015; Hudson et al. 1995; Bellani et al. 2016; Meyer and Prescott 2024). Sepsis is defined as a dysregulated host response to infection leading to life‐threatening organ injury (Singer et al. 2016). Of the various clinical risk factors for ARDS, sepsis is associated with both the highest incidence and highest mortality of ARDS (Hudson et al. 1995; Eisner et al. 2001). One study comparing sepsis‐related ARDS to ARDS from other causes found that patients with sepsis‐related ARDS had worse clinical outcomes including severity of hypoxemia, severity of illness, ventilator duration and 60‐day mortality (Sheu et al. 2010). In a multivariate analysis accounting for severity of illness and comorbidities, sepsis itself was not independently associated with mortality, suggesting that the poor outcomes in sepsis‐related ARDS may be driven by the severity of multiorgan failure and underlying physiologic reserve (Sheu et al. 2010). Death from ARDS most often occurs from refractory multiorgan failure and shock rather than respiratory failure itself (Stapleton et al. 2005; Ketcham et al. 2020).

Sepsis‐associated AKI (SA‐AKI) is AKI that occurs within 7 days of sepsis (Zarbock et al. 2023; Poston and Koyner 2019). Outcomes including mortality are worse for patients with AKI from sepsis compared with AKI from other causes (Mehta et al. 2011, 2016). AKI can also precede sepsis and may increase susceptibility to it (Mehta et al. 2011). The pathologic mechanisms of SA‐AKI are multifactorial and still being elucidated. In SA‐AKI, the classic understanding was that overt macrocirculatory dysfunction (via hemodynamic instability) was the driver of injury, but newer research has established that microcirculatory dysfunction is also a major contributor leading to tissue damage via patchy hypoxia and localized ischemia–reperfusion injuries even when global renal perfusion is maintained (Zafrani et al. 2015; Post et al. 2017; Pais et al. 2024). Microcirculatory dysfunction in SA‐AKI is driven by inflammation, endothelial dysfunction, microvascular thrombi and oxidative stress (Zafrani et al. 2015; Post et al. 2017). Additional mechanisms of SA‐AKI include metabolic reprogramming, mitochondrial dysfunction, interstitial edema and renin‐angiotensin‐aldosterone system dysfunction (Post et al. 2017; Zarbock et al. 2023; Pais et al. 2024).

In animal studies, sepsis is typically modeled with three approaches: exogenous toxin administration (e.g., lipopolysaccharide, LPS), exogenous single‐pathogen administration at a particular site (e.g., intratracheal pneumococcus instillation) or disruption of the animal's internal barriers creating a polymicrobial infection (e.g., cecal ligation and puncture, CLP) (Table 1) (Doi et al. 2009). CLP in mice generally produces a sepsis‐like disorder, yet lung injury and kidney injury are challenging to consistently replicate with this model (Doi et al. 2009; Hukriede et al. 2022). An alternative to CLP is cecal slurry in which fecal contents from one animal are injected into the peritoneum of another animal; this method may permit greater titration of the polymicrobial inoculum and is thought to avoid a more unpredictable inflammatory response that can occur in the surgical CLP approach (Kannan et al. 2024).

**TABLE 1:** Selected animal models of organ injury described in lung‐kidney crosstalk literature.

Disease Model	Description	Considerations	Examples
Acute kidney injury			Reviewed in (Hukriede et al. 2022)
Ischemia	Bilateral or unilateral cross clamping of the renal pedicle	– Clear timing – Injury very severe	Mice (Deng et al. 2004, Molls et al. 2006, Zarbock et al. 2006, Hassoun et al. 2007, Grigoryev et al. 2008, Klein et al. 2008, Awad et al. 2009, Hassoun et al. 2009, Andres‐Hernando et al. 2011, Feltes et al. 2011, Ahuja et al. 2012, Allam et al. 2012, Altmann et al. 2012, White, Cui, et al. 2012a, White, Santora, et al. 2012b, Andres‐Hernando et al. 2017, Nakazawa et al. 2017, Nakazawa et al. 2018, Hayase et al. 2020, Ambruso et al. 2021, Hepokoski et al. 2021, Khamissi et al. 2022, Komaru et al. 2025) Rats (Kramer et al. 1999, Rabb et al. 2003, Sharfuddin et al. 2009, White, Cui, et al. 2012a, Ma and Liu 2013)
Nephrectomy	Surgical removal of one or both kidneys	– Clear timing – Lung injury less consistent	Mice (Zarbock et al. 2006, Hassoun et al. 2007, Hoke et al. 2007, Klein et al. 2008, Ishii et al. 2010, Feltes et al. 2011, Rossaint et al. 2011, Ahuja et al. 2012, Andres‐Hernando et al. 2012, Doi et al. 2014) Rats (Yabuuchi et al. 2016)
Oxidative stress	Ex‐vivo exposure of renal tubular epithelial cells to reactive oxygen species	– Cell target clear – In vivo response may be different	Human cells ex‐vivo (DeWolf et al. 2024)
Lung injury/ARDS			Reviewed in (Matute‐Bello et al. 2008)
Ventilator Induced	Injurious vs non‐injurious volumes or pressures given via mechanical ventilation	– Usually in combination with another injury model – Size of animal may impact feasibility of	Mice (Hepokoski et al. 2017) Rabbits (Imai et al. 2003) Rats (Felix et al. 2022) Dogs (Hoag et al. 2008)
Acid Aspiration	Inhalation of acid (e.g., HCl) epithelial injury	– May not reflect gastric aspiration contents	Rabbits (Imai et al. 2003) Dogs (Hoag et al. 2008)
Sepsis			Reviewed in (Matute‐Bello et al. 2008, Doi et al. 2009)
Exogenous toxin	E.g., lipopolysaccharide (LPS)	– Route of delivery (i.e., intratracheal, intraperitoneal) – Dose	General sepsis (Czaikoski et al. 2016, Okeke et al. 2020) Renal injury (Allam et al. 2012, Ni et al. 2021) Lung injury (Ma and Liu 2013, Felix et al. 2022, Kim et al. 2022)
Single pathogen	E.g., *Pseudomonas aeruginosa* or *Streptococcus*	– Route of delivery – Bacterial load	(Lange et al. 2010, Singbartl et al. 2011, Ni et al. 2021, Kim et al. 2022)
Polymicrobial	E.g., Cecal ligation and puncture (CLP)	– Lung and kidney injury after CLP historically less reproducible compared to other sepsis model – Length of barrier disruption equivalent to dose of bacterial load	Renal injury (Czaikoski et al. 2016, Ni et al. 2021) Lung injury (Hepokoski et al. 2017)

## 3 Effects of Kidney Injury on the Lung

### 3.1 Models of Kidney Injury

The effects of AKI on pulmonary pathophysiology have been demonstrated in clinical studies, but animal models of AKI have provided the most robust source of mechanistic insights (Table 1). Animal models of lung injury following AKI from ischemia–reperfusion injury (Deng et al. 2004; Klein et al. 2008; Ahuja et al. 2012; Andres‐Hernando et al. 2011; White, Santora, et al. 2012b) or nephrectomy (Klein et al. 2008; Hoke et al. 2007) are the most prevalent AKI models in the lung crosstalk literature. Injury to the kidneys can be initiated prior to measurements of lung pathophysiology, making the temporal sequence of crosstalk clear. Another strength of these models is the lack of confounding relationships due to shared risk factors seen in other models (e.g., sepsis) (Hukriede et al. 2022).

Ischemia–reperfusion injury has been the most frequently used model in the crosstalk literature (referred to here as ischemic AKI). Ischemic AKI in mice is typically conducted via transient renal pedicle clamping with sham surgical controls and is thought to emulate macrovascular injury, that is, a reduction in renal blood flow during hypotensive episodes or during surgical procedures such as cardiac bypass (Hukriede et al. 2022). This model tends to create histologic abnormalities that may be more severe than what is commonly seen in adult ICU patients (Hukriede et al. 2022). Nephrectomy models involve surgical kidney removal, and although lung injury has been demonstrated after nephrectomy (Klein et al. 2008; Hoke et al. 2007; Doi et al. 2014; Ishii et al. 2010) it has been less reproducible compared with ischemia models (Khamissi et al. 2022; Hassoun et al. 2007). Comparisons between ischemia and nephrectomy models allow evaluation of both direct tissue injury and decreased renal clearance in ischemia models contrasted with an isolated drop in renal clearance in nephrectomy models (Faubel and Edelstein 2016). For instance, impaired clearance of cytokines or uremic toxins after nephrectomy could contribute to lung inflammation even in the absence of direct renal tissue damage (Hoke et al. 2007; Lowenstein and Nigam 2021; Andres‐Hernando et al. 2012). Overall, however, the extent of injury and lung inflammation after AKI by nephrectomy is less severe than ischemic AKI in mice (Faubel and Edelstein 2016; Hassoun et al. 2007). Direct comparisons between ischemia and nephrectomy models reveal different biologic responses, and suggest that signaling from ischemic kidneys may trigger lung inflammation to a greater degree compared with loss of renal function alone (Hassoun et al. 2007). Standard animal models of sepsis have been used to characterize SA‐AKI (Doi et al. 2009), but crosstalk as an explicit outcome has been infrequently assessed using these models.

**FIGURE 1:**
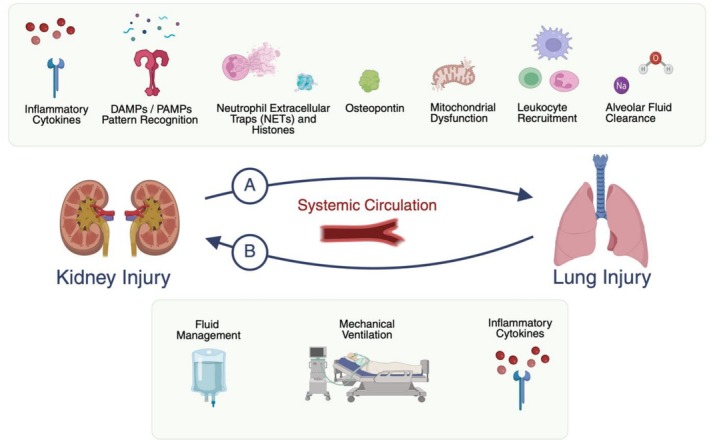
Key mediators and mechanisms of lung‐kidney cross talk after organ injury. (A) Experimental evidence of lung injury after kidney injury supports a wide range of mediators: Inflammatory cytokines including IL‐6, IL‐8; the tumor necrosis factor pathway (TNF‐α/TNFR1) activation; pattern recognition receptors (PRRs) such as toll‐like receptor 4 (TLR4) which bind to danger‐associated molecular patterns (DAMPs) and pathogen‐associated molecular patterns (PAMPs); creation of neutrophil extracellular traps (NETs) and circulating histones; mitochondrial dysfunction and metabolic reprogramming; recruitment of macrophages, neutrophils and T‐cells to the lung; and impaired alveolar fluid clearance through effects on water and sodium transport. (B) Experimental evidence of kidney injury after lung injury includes inflammatory biomarkers such as IL‐6, soluble tumor necrosis factor (sTNFR‐I and sTNFR‐II), plasminogen activator inhibitor 1 (PAI‐1); effects of injurious mechanical ventilation; and consequences of fluid administration including negative effects of volume overload.

**FIGURE 2:**
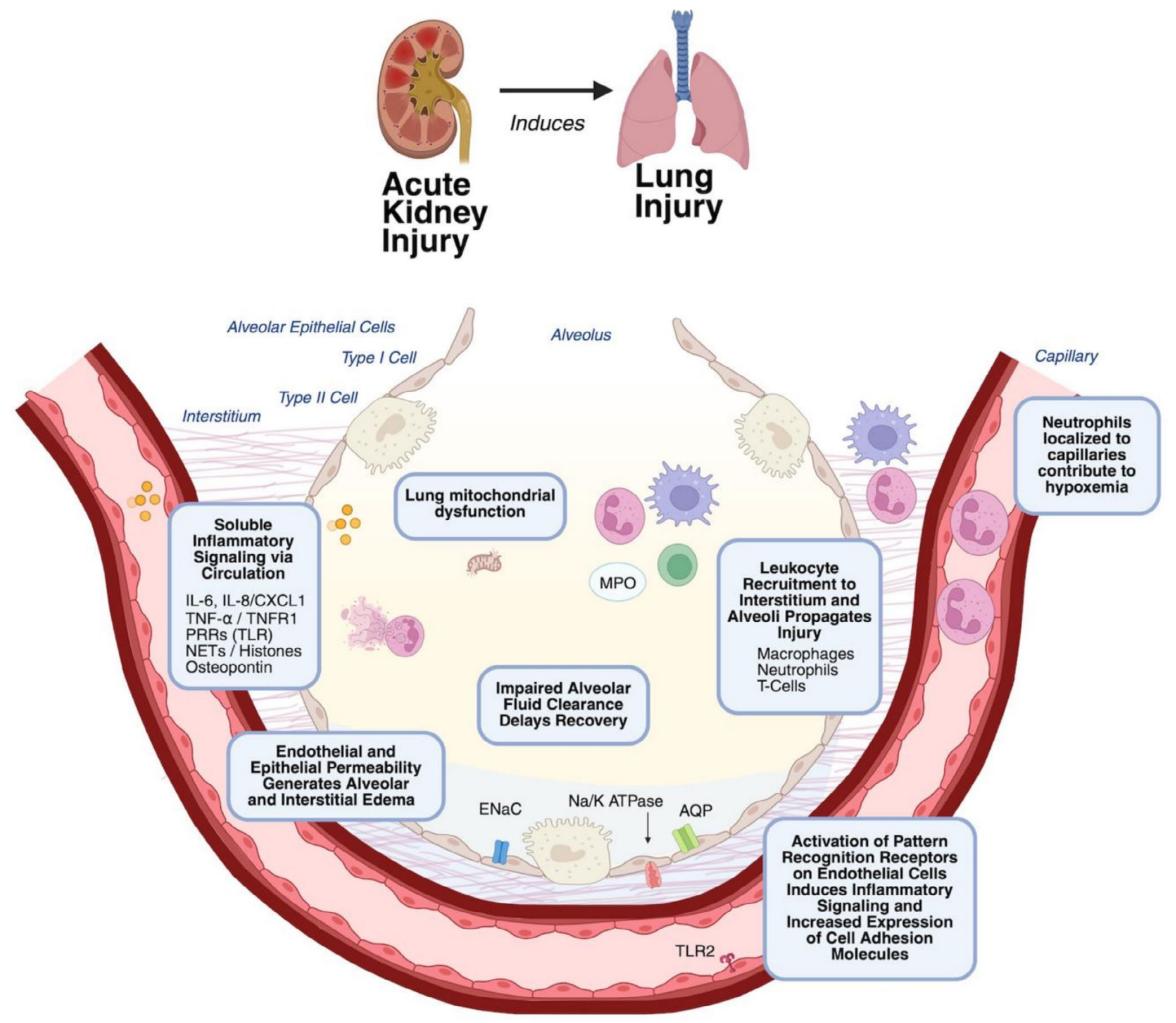
Mechanisms of alveolar injury after experimentally generated acute kidney injury (AKI). A large range of soluble mediators from kidney injury pass through the pulmonary circulation to generate an inflammatory response in the lungs which is then further amplified due to local damage. Soluble mediators include the cytokines IL‐6, IL‐8 or CXCL1; TNF‐α/TNFR1; pattern recognition receptors including toll‐like receptors (TLRs); neutrophil extracellular traps (NETS); and circulating histones; and renal tubular cell‐derived osteopontin. Leukocytes are detected in the lungs rapidly following AKI including macrophages, neutrophils and T‐cells. Neutrophil enzymes including myeloperoxidase (MPO) are increased. Activation of pattern recognition receptors on pulmonary endothelial cells increases inflammatory signaling and the expression of cell adhesion molecules. Neutrophils localizing to pulmonary capillaries reduce blood flow and contribute to reduced gas exchange. The local inflammatory response in the lung creates endothelial and epithelial permeability resulting in non‐hydrostatic pulmonary edema. AKI downregulates alveolar fluid clearance through effects on the epithelial sodium channel (ENaC), the sodium‐potassium ATPase pump (N/K ATPase) and aquaporins in alveolar epithelial cells resulting in slowed resorption of alveolar fluid. Mitochondrial dysfunction of lung tissue is also observed following AKI.

After AKI induced by ischemia, nephrectomy or sepsis, lung histology demonstrates neutrophilic inflammation with edema consistent with lung injury (Hassoun et al. 2007, 2009). Specific pathways have been implicated in the pathogenesis of AKI in these models which is reviewed in detail in the following sections (Figure 1). Circulating soluble mediators are thought to be a primary route of communication between the kidney and lungs in AKI‐derived lung injury (Figure 2) (Faubel and Edelstein 2016; Komaru et al. 2024). Ischemic AKI in mice induced gene expression changes in lung tissue within inflammatory, immune and pro‐apoptotic pathways compared with sham procedure (Hassoun et al. 2007, 2009). The pulmonary endothelium is likely a key target of circulating mediators after injury and is central to the development of lung injury (Huppert et al. 2019; Feltes et al. 2011). Pulmonary endothelial cells isolated from mice after ischemic AKI also demonstrate increased gene expression related to inflammation and programmed cell death (Feltes et al. 2011). Communication from the kidney to the lung may not only occur via the circulation. Crosstalk between the peripheral nervous system and local immune cells in the injured kidney may activate central nervous system pathways, which in turn could influence distant organs including the lungs. Neuro‐immune interactions after AKI are well reviewed in reference (Herrlich 2022).

### 3.2 Leukocyte Recruitment

Alveolar macrophages and neutrophils are detected in the lung after AKI and are key contributors to organ crosstalk (Komaru et al. 2024). Alveolar macrophages accumulate early after AKI and participate in the initial response to injury by recruiting neutrophils. Interstitial neutrophils and macrophages are detected early after AKI in mice (Klein et al. 2008) and interstitial macrophages remain significantly elevated through Day 5 after ischemic AKI (Khamissi et al. 2022). Inhibition of macrophage activation attenuates lung injury after ischemic AKI in rats (Kramer et al. 1999). Depletion of either systemic or alveolar macrophages blunts the increase in neutrophil recruitment and activity that was detected after ischemic AKI (Altmann et al. 2012). T‐cells are also recruited to the lung early after ischemic renal injury in mice and participate in apoptosis (Faubel and Edelstein 2016).

Neutrophils are increased in the lung at 2 h following ischemic AKI (Awad et al. 2009), peak at Day 1 and remain significantly elevated until Day 5 compared with sham (Khamissi et al. 2022). Increased neutrophil activity by enzyme assays of neutrophil myeloperoxidase (MPO) (Klein et al. 2008; Doi et al. 2014; Altmann et al. 2012, 2017; Hayase et al. 2020) and neutrophil elastase (Doi et al. 2014; Ishii et al. 2010) can be detected after ischemic AKI and nephrectomy. A comparison in mice with ischemic AKI induced lung injury and mice with direct lung injury from LPS administration revealed that after AKI, lung neutrophils were largely restricted to the intravascular space whereas after direct lung injury the majority of neutrophils extravasated into the interstitium and alveoli (Komaru et al. 2025). Furthermore, this recruitment of neutrophils to lung capillaries can induce hypoxemia via a reduction in alveolar blood flow after ischemic AKI in mice, while hypoxemia from classical lung injury is primarily driven by reduced gas exchange from interstitial and alveolar edema (Komaru et al. 2025). Reduced neutrophil deformability may contribute to the accumulation of neutrophils in pulmonary capillaries after AKI, and mechanisms of neutrophil recruitment likely differ between remote and direct lung injury (Komaru et al. 2025).

It remains unclear if neutrophils recruited to the lung after AKI are similar to populations of neutrophils that propagate direct lung injury. Though AKI increases the quantity of pulmonary neutrophils, it may alter their movement and function. Neutrophil migration, particularly slow rolling, is impaired by AKI in patients with sepsis (Rossaint et al. 2011) and murine AKI (nephrotoxic and rhabdomyolysis models) (Rossaint et al. 2011; Singbartl et al. 2016). Resistin, a uremic toxin found in mice and patients after AKI, contributes to impaired neutrophil migration (Singbartl et al. 2016) and may also inhibit the bactericidal activity of neutrophils (Miller et al. 2019). In a two‐hit model where mice received AKI (ischemic or nephrectomy) followed by an acid‐induced lung injury (an aspiration pneumonitis model), mice with both types of AKI exhibited less hypoxemia after lung injury compared with mice that underwent a sham AKI procedure before lung injury (Zarbock et al. 2006). Reconstitution experiments indicated that uremic neutrophils are a key mediator of this differential effect. After inducing AKI with nephrectomy, neutrophil depletion was performed. Mice then received either uremic neutrophils, non‐uremic neutrophils or uremic plasma before lung injury. Neutrophil‐depleted mice with AKI that received uremic neutrophils had reduced lung injury compared with those given non‐uremic neutrophils or uremic plasma. The authors hypothesized that the effect of uremia on neutrophil function may be due to the reduction of the cell adhesion molecule L‐selectin (Zarbock et al. 2006). These results highlight the complexity of crosstalk mechanisms and the importance of both model timing and selection. Though most examples in the literature demonstrated worsening lung injury after AKI, in this example AKI preceding a second hit of aseptic lung injury had a protective effect on pulmonary inflammation.

### 3.3 Inflammatory Cytokines and Chemokines

Inflammatory signaling following AKI is an important driver of remote lung injury. The soluble mediators interleukin‐6 (IL‐6) and interleukin‐8 (IL‐8) are major contributors to lung injury after AKI (Klein et al. 2008; Ahuja et al. 2012; Hoke et al. 2007; Grigoryev et al. 2008; Liu et al. 2009). Serum IL‐6 is elevated early (2 h) and late (24 h+) after AKI in both ischemic and bilateral nephrectomy in mice compared with sham (Klein et al. 2008; Ahuja et al. 2012; Hoke et al. 2007; Hayase et al. 2020; Grigoryev et al. 2008; Nakazawa et al. 2017). Transcriptomics of lung and kidney tissue after ischemic AKI in mice demonstrated an increase in a large number of inflammatory genes compared with sham, specifically identifying the pro‐inflammatory IL‐6 pathway as differentially expressed (Grigoryev et al. 2008). CXCL1 (C‐X‐C motif chemokine ligand 1), also called KC (keratinocyte‐derived chemokine), is a functional murine homologue of IL‐8 and is considered a neutrophil chemokine (Molls et al. 2006; Hol et al. 2010; Matute‐Bello et al. 2008). In mice, CXCL1 increases in serum early after ischemic AKI (Hoke et al. 2007; Molls et al. 2006) and is elevated in lung tissue in both ischemic AKI and bilateral nephrectomy (Klein et al. 2008; Ahuja et al. 2012). Inhibition of CXCL1 reduced lung inflammation after both types of AKI (Ahuja et al. 2012).

IL‐6 in AKI may potentiate pulmonary injury through the action of CXCL1 to recruit neutrophils, which are a key mediator of lung injury (Huppert et al. 2019). After both ischemic AKI and bilateral nephrectomy in wild‐type mice, neutrophil MPO and the neutrophil chemokine CXCL1 were increased in lung tissue compared with sham procedure (Klein et al. 2008). The mice had increased pulmonary capillary permeability and pulmonary histology consistent with lung injury (Klein et al. 2008). After AKI in IL‐6 deficient mice, neutrophil MPO activity, CXCL1 levels and pulmonary capillary leak were still increased, but to a significantly lesser extent than in wild‐type mice (Klein et al. 2008). Lung injury markers were also attenuated in wild‐type mice given an anti‐IL‐6 antibody after AKI. Additional evidence suggests that circulating IL‐6, rather than lung‐derived IL‐6, mediates injury post AKI through CXCL1. (Ahuja et al. 2012). Thus, IL‐6 influences lung injury after AKI caused by both ischemia and nephrectomy, and functions in part through the action of chemokines such as IL‐8/CXCL1 to recruit neutrophils.

Counter‐inflammatory responses via interleukin‐10 (IL‐10) influence the degree of lung injury after AKI. IL‐10 pathway genes are upregulated after ischemic AKI (Grigoryev et al. 2008) and serum IL‐10 is increased after both ischemic AKI and bilateral nephrectomy in mice (Andres‐Hernando et al. 2011, 2012; Hayase et al. 2020). Although IL‐6 is an inflammatory cytokine, it may also be necessary for the initiation of a counter‐inflammatory response mediated by IL‐10. Production of IL‐10 was reduced after AKI in IL‐6 deficient mice (Andres‐Hernando et al. 2017). Mice that received both splenectomy and ischemic AKI had increased IL‐6 and increased markers of lung injury compared with mice that had AKI alone (Andres‐Hernando et al. 2011). This was due to the lack of splenic production of IL‐10, which attenuates lung injury after AKI (Andres‐Hernando et al. 2011, 2017). CD4‐lymphocytes may also participate in the counter‐inflammatory response after ischemic AKI: CD4‐knockout mice had reduced IL‐10 production and increased lung MPO activity relative to wild‐type mice (Andres‐Hernando et al. 2017). Overall these results indicate that IL‐10 has a counter‐inflammatory role in lung injury induced by AKI which is concordant with its known function in the immune response to injury.

The tumor necrosis factor (TNF) and tumor necrosis factor receptor (TNFR) pathways, with signaling through nuclear factor kappa B (NF‐κB), also contribute to pulmonary injury following AKI. Ischemic AKI is associated with lung endothelial cell apoptosis in rats and mice which is mediated through TNF‐α/TNFR (White, Cui, et al. 2012a). After ischemic AKI in mice, circulating TNF‐α is increased (White, Santora, et al. 2012b; Hayase et al. 2020; Nakazawa et al. 2017), expression of TNFR pathway genes is increased (Hassoun et al. 2009) and TNFR‐1 is increased in lung tissue (White, Santora, et al. 2012b; Hassoun et al. 2009). NF‐κB and caspase 3 are also increased in lung after ischemic AKI (Deng et al. 2004; White, Santora, et al. 2012b). TNFR‐1‐knockout mice had reduced pulmonary apoptosis and decreased NF‐κB expression compared with wild‐type mice after ischemic AKI (White, Santora, et al. 2012b; Hassoun et al. 2009). TNF‐α inhibition reduced NF‐κB levels, pulmonary apoptosis and pulmonary edema in mice after ischemic AKI (White, Santora, et al. 2012b).

Together, these data suggest that ischemic kidney injury induces pulmonary TNFR‐1 signaling that impacts pulmonary inflammation via the NF‐κB pathway.

### 3.4 Pattern Recognition Receptors (PRRs)

Pattern recognition receptors (PRRs), such as Toll‐like receptors (TLRs), are key components of the innate immune response and likely contribute to interorgan crosstalk during kidney injury. PRRs detect both pathogen and tissue damage through pathogen‐associated molecular patterns (PAMPs) and danger‐associated molecular patterns (DAMPs). A range of molecules can activate PRRs including nucleic acids, membrane components, and peptides from pathogens. PRR activation contributes both to the pathogenesis of sepsis and multiorgan injury in critical illness (Takeuchi and Akira 2010; Salomao et al. 2008; Tolle and Standiford 2013). For instance, lipopolysaccharide (LPS), a component of the cell wall of gram‐negative bacteria, often used to model sepsis, functions through TLR4 activation (Poltorak et al. 1998). TLR2 and TLR4 are upregulated in the kidney in both ischemic and septic kidney injury models, contributing to local inflammatory responses and organ dysfunction (Valles et al. 2014).

TLR receptor signaling is also involved in the initiation of lung injury following AKI. C3H/HeJ mice have a mutation in TLR4 that impedes downstream signaling through this receptor (Poltorak et al. 1998). Early after injury via bilateral nephrectomy, C3H/HeJ mice with impaired TLR4 signaling exhibited less lung neutrophil infiltration, lower plasma and lung neutrophil elastase activity, lower lung MPO activity and reduced vascular permeability compared with control mice undergoing the same injury (Doi et al. 2014). Next the effects of high‐mobility group protein B1 (HMGB1), an agonist of TLR‐4, were examined in the C3H/HeJ mice by giving an anti‐HMGB1 neutralizing antibody. After bilateral nephrectomy, the HMGB1 blockade reduced lung neutrophil activity in the control mice but not the TLR4‐impaired mice, indicating that TLR4 is necessary for the propagation of injury. After ischemic AKI in contrast, HMGB1 blockade reduced neutrophil activity in both the TLR4‐impaired and control mice, indicating an effect of HMGB1 on lung injury after AKI independent of TLR4. These results suggest that there are different mechanisms of injury after AKI on the HMGB1–TLR4 pathway and the mechanisms may be influenced by the type of renal injury.

TLR signaling propagates lung injury through action at pulmonary endothelial cells after AKI. DeWolf and team developed an ex vivo model of lung injury following AKI from oxidative stress to study the mechanistic role of DAMPs and PRRs (DeWolf et al. 2024). AKI was modeled via human renal tubular epithelial cells that were exposed to reactive oxygen species to generate a necrotic supernatant containing DAMPs known to interact with PRRs. Human pulmonary microvascular endothelial cells were then exposed to this DAMP‐containing supernatant from the injured renal cells. Pulmonary endothelial cells differentially expressed genes related to pathways of TLR and another PRR, NOD‐like receptor (NLR), with upregulation of TLR2 and NOD2. Signaling of PRRs was indicated by evidence of their downstream effects in the mitogen‐activated protein kinase (MAPK) and NF‐κB pathways. Pulmonary endothelial cells exposed to necrotic supernatant also produced increased levels of inflammatory cytokines and chemokines (IL‐6, IL‐8, MCP‐1 and GM‐CSF). Application of NOD1 and NOD2 inhibitors attenuated IL‐6 and MCP‐1 production, a finding that may be particularly important given the relationship between IL‐6 and lung injury described in the prior section. The exposed pulmonary endothelial cells had an increase in expression of the cell adhesion molecule E‐selectin relative to controls and demonstrated increased permeability in a cell culture permeability model.

### 3.5 Neutrophil Extracellular Traps and Histones

Neutrophil extracellular traps (NETs) and circulating histones have been implicated in a wide range of inflammatory diseases, including sepsis, ARDS and SA‐AKI (Scozzi et al. 2022; Ni et al. 2021; Xu et al. 2009; Abrams et al. 2013; Papayannopoulos 2018), and have been studied as potential mechanisms of lung injury after kidney injury (Nakazawa et al. 2018). NETs are sections of chromatin and proteins, including anti‐microbial compounds and proteases, which are excreted by neutrophils (Papayannopoulos 2018). NETs trap and kill extracellular pathogens as part of the innate immune response, but have also been found in sterile inflammation (Papayannopoulos 2018; Brinkmann et al. 2004). Importantly, NETs can cause collateral damage to adjacent tissues through direct injury (e.g., neutrophil elastase) or via downstream signaling as DAMPs (e.g., histones).

Histones are present in the released chromatin of NETs but are also released from other dying or injured cells (Holdenrieder and Stieber 2009). Though histone‐mediated damage is a component of lung–kidney crosstalk during injury, this mechanism is nonspecific. For instance, histones are mechanistically linked to trauma‐associated lung injury (Abrams et al. 2013). Histones are recognized as DAMPs, and downstream signaling through TLRs may be one mechanism of both NET function and collateral organ toxicity (Nakazawa et al. 2017, 2018). In cell culture studies histones are released by necrotic renal tubular epithelial cells (Allam et al. 2012) and can induce human pulmonary endothelial cells to release cytokines (Abrams et al. 2013), and also increase endothelial cell permeability (Kim et al. 2022). These effects of histones are dependent on TLR signaling (Allam et al. 2012; Kim et al. 2022). Blocking NET formation in mice reduces both lung injury and kidney injury induced by sepsis in LPS and cecal ligation and puncture models (Ni et al. 2021; Okeke et al. 2020; Czaikoski et al. 2016). These data suggest that histones from NETs and other sources may mediate injury in different tissues and may represent a shared mechanism of lung and kidney injury after sepsis.

Additional direct experimental evidence supports a role of NETs in lung injury after ischemic AKI in mice. Nakazawa and colleagues demonstrated that after ischemic AKI in mice, NETs were detected locally in the kidney, circulating in plasma, and remotely in the lung tissue compared with mice undergoing a sham procedure (Nakazawa et al. 2017). In the lung, NETs co‐localized with areas of cellular apoptosis by TUNEL staining indicating local cytotoxicity (Nakazawa et al. 2017). Inhibition of either NETs or anti‐histone antibodies reduced the degree of post‐AKI neutrophil infiltration and apoptosis in lung tissue (Nakazawa et al. 2017). Further support for the role of histones in propagating lung injury after AKI comes from a study utilizing thrombomodulin, which is thought to bind to circulating histones and reduce renal injury in a rat ischemic AKI model (Sharfuddin et al. 2009). Mice pretreated with recombinant thrombomodulin before ischemic AKI had decreased levels of pulmonary histone accumulation, NET formation, vascular permeability, MPO activity and neutrophil infiltration relative to mice who underwent ischemic AKI but did not receive thrombomodulin (Hayase et al. 2020).

### 3.6 Osteopontin

Circulating osteopontin (OPN), released from kidney tubular cells, has been identified as a novel mediator of AKI‐induced lung injury. Khamissi et al. used a ligand‐receptor pairing analysis of single‐cell RNA‐seq data to identify molecules relevant to lung‐kidney cross talk in ischemic kidney injury (Khamissi et al. 2022). The investigators specifically targeted soluble ligands expressed in kidney cells that had related receptors in lung cells. At baseline before injury, OPN was identified as a key lung‐kidney pairing molecule with production localized to distal renal tubule cells. The significance of the OPN lung‐kidney pairing increased after injury and OPN was identified from a larger number of renal cell types. OPN binds with the CD44 receptor in non‐immune and immune cells in the lungs. OPN (also called secreted phosphoprotein‐1, SPP‐1) is named for its role in bone mineralization, but it has broader immune system functions including as a chemotactic agent for macrophages (Ashkar et al. 2000; Wang and Niu 2024; Jia et al. 2024). In the same study, fluorescently labeled OPN co‐localized with interstitial macrophages and alveolar macrophages in lung tissue after ischemic AKI (Khamissi et al. 2022). OPN gene expression increased early after AKI in renal cells, but not lung cells, and corresponded with OPN protein detection in serum after injury. OPN inhibition by either anti‐OPN antibody or global OPN knockout substantially decreased this ischemic AKI‐induced lung injury in mice. Transplantation of ischemic kidneys from wild‐type mice into OPN‐knockout recipients caused lung injury whereas transplantation of ischemic kidneys from OPN‐knockout mice into wild‐type recipients did not, indicating that kidney‐derived OPN is essential for crosstalk (Khamissi et al. 2022). Mice with a mild degree of AKI insufficient to generate lung injury were injected with OPN and subsequently developed lung injury that was comparable to the injury seen in mice with severe AKI without OPN injection. Importantly, OPN injection into uninjured mice did not result in inflammatory changes in the lung, indicating that OPN alone is not sufficient for lung injury generation without kidney injury. Together these results suggest that circulating OPN generated by the injured kidney is a crucial mechanism in the development of lung injury post‐AKI.

These animal data are supported by clinical studies examining OPN, but notably the role of OPN in mediating organ crosstalk may not be exclusive to kidney and lung interactions (Zhao et al. 2018; Kahles et al. 2014; Castello et al. 2019). In a secondary analysis of a sepsis clinical trial, Day 1 plasma OPN levels were associated with having septic shock, increased multiorgan dysfunction by SOFA score, increased creatinine levels and mortality (Carbone et al. 2020). Decreasing levels of OPN over the first 7 days following hospitalization were associated with increased survival (Carbone et al. 2020).

### 3.7 Metabolic Alterations and Mitochondrial Dysfunction

Animal models have provided evidence that AKI results in remote disruptions of metabolic pathways in lung tissue. Metabolomics assays have identified distinct metabolic profiles between ischemic AKI and sham AKI in mice, which were consistent with a shift away from typical oxidative phosphorylation and toward alternative sources of energy production (Ambruso et al. 2021). After ischemic AKI, mice showed depletion of lung glutathione, a critical antioxidant, consistent with oxidative stress (Ambruso et al. 2021). Lung ATP levels are decreased in mice at 4 and 24 h after ischemic AKI compared with sham surgery (Ambruso et al. 2021).

Mitochondrial dysfunction may play a crucial role in the initiation of AKI (Hepokoski and Singh 2022). Hepokoski and colleagues have identified that mitochondrial dysfunction and the production of mitochondrial DAMPs (mtDAMPs) may participate in lung injury following ischemic AKI (Hepokoski et al. 2021). Metabolomic profiles of mouse lung tissue were significantly different at 4 h following ischemic AKI compared with a sham procedure in a pattern consistent with impaired fatty acid oxidation (FAO) of mitochondria. Elevated levels of extracellular mtDAMPs were detected in both plasma and BAL fluid of the injured mice. In healthy mice, an intraperitoneal injection of a solution containing mtDAMPs isolated from ischemic kidney tissue elicited a change in lung metabolism consistent with impaired phospholipid metabolism and FAO. In other contexts, mtDAMPs have been shown to elicit lung injury in rats (Zhang et al. 2010).

### 3.8 Fluid Balance and Alveolar Fluid Clearance

Fluid overload from renal failure is associated with increased mortality for critically ill patients with AKI (Bouchard et al. 2009). Fluid overload can directly impact pulmonary function and can be particularly challenging in the setting of respiratory failure. Fluid accumulation from renal causes can contribute to both cardiogenic pulmonary edema via an increase in lung vascular hydrostatic pressure and can exacerbate non‐cardiogenic pulmonary edema in ARDS through increased permeability of damaged endothelium and epithelium. In both instances, active sodium ion transport is required for vectorial clearance of fluid from the alveoli to restore normal gas exchange (Huppert et al. 2019). Not only can AKI generate volume overload that contributes to pulmonary edema, but experimental models indicate that AKI directly disrupts alveolar fluid clearance through effects on sodium and water transport. Both ischemic AKI and bilateral nephrectomy in rats resulted in downregulation of lung sodium channels, which are critical to alveolar fluid clearance, and lung aquaporins, which facilitate movement of water across the epithelium (Huppert et al. 2019; Rabb et al. 2003; Yabuuchi et al. 2016; Ma and Liu 2013).

### 3.9 Organ Support: Mechanical Ventilation and Renal Replacement Therapy (RRT)

Mechanical ventilation is frequently required for patients with respiratory failure or ARDS. Outcomes are worse for ventilated ICU patients with AKI compared with those without AKI and the presence of AKI may negatively impact ventilatory parameters (Vemuri et al. 2022). A retrospective cohort study of 4484 mechanically ventilated patients found that patients with AKI had increased plateau pressures, higher driving pressures and lower compliance compared with patients without AKI (Vemuri et al. 2022). Interestingly, this finding persisted even for patients with a negative cumulative fluid balance, suggesting that the effects of AKI on ventilation parameters may not be a result of volume overload alone.

Renal replacement therapy (RRT) is an essential therapy for critically ill patients with severe AKI or ESRD. Overall, critically ill patients with AKI severe enough to require RRT have worse clinical outcomes (Mehta et al. 2016). The primary goal of RRT is to treat abnormalities related to impaired renal clearance: correcting acidosis, uremia, electrolyte or fluid imbalances. The timing of RRT initiation in critically ill patients has been extensively studied: currently there is insufficient evidence to support an early RRT initiation approach compared with a delayed RRT approach for ICU patients generally and ARDS patients specifically (Ostermann et al. 2025; Gaudry et al. 2018). An interest in testing early RRT strategies stemmed in part from the hypothesis that AKI is associated with reduced renal clearance of cytokines, and dialysis might remove inflammatory compounds from the blood that propagate renal and multiorgan injury (Komaru et al. 2024; Lowenstein and Nigam 2021; Andres‐Hernando et al. 2012). At present there is no evidence to support the use of RRT for cytokine clearance in lung or kidney injury.

## 4 Effects of Lung Injury on the Kidney

### 4.1 Models of Lung Injury

A range of experimental animal models of lung injury has been developed to recapitulate the pathologic consequences of lung injury (Table 1). Models of lung injury function by exposing animals to a known underlying risk factor for ARDS in humans. Sepsis as a cause of lung injury is modeled using live bacteria (pulmonary or extrapulmonary), via cecal ligation and puncture or through the administration of LPS (Matute‐Bello et al. 2008). Additional models include acid aspiration, hyperoxia, mechanical ventilation, saline lavage for surfactant depletion and ischemia reperfusion injury (Matute‐Bello et al. 2008).

In contrast to the extensive experimental literature studying lung injury after AKI, a limited number of studies have explicitly tested renal injury following induced lung injury in animals. Both kidney injury and lung injury have been demonstrated after a variety of sepsis models, consistent with the understanding that sepsis is a systemic disorder that drives multiple organ dysfunction (Hukriede et al. 2022; Matute‐Bello et al. 2008). Compared with sham injury, lung injury in sheep via smoke inhalation followed by *Pseudomonas aeruginosa* instillation resulted in a drop in microvascular blood flow in the renal cortex (Maybauer et al. 2010), increased serum creatinine and decreased urine output (Lange et al. 2010). Compared with sham, induction of bacterial pneumonia with *Pseudomonas aeruginosa* in mice caused increases in serum creatinine, serum cystatin C, and urinary NGAL (tubular injury marker) consistent with AKI that was neutrophil independent and occurred despite similar renal perfusion between the groups (Singbartl et al. 2011). Renal injury has also been noted in models combining injurious ventilation and either acid (Imai et al. 2003) or LPS (Felix et al. 2022) induced lung injury with evidence of increased serum creatinine (Imai et al. 2003), renal tubular cell apoptosis (Imai et al. 2003) and increased IL‐6 and NGAL gene expression in kidney tissue (Felix et al. 2022).

### 4.2 Inflammatory Cytokines

The prior sections illustrated that inflammatory cytokines are a key mediator of lung injury after AKI, and clinical evidence supports the notion that they also mediate AKI after lung injury. For instance, a secondary analysis of data from an ARDS clinical trial examined the role of plasma biomarkers on the risk for AKI among 876 patients with ARDS but without ESRD (Liu et al. 2007). Investigators found that 209 (24%) developed AKI, defined as an increase in creatinine of at least 50% above baseline, in the first four days following enrollment. Baseline plasma inflammatory biomarkers were tested for association with the development of AKI in adjusted regression models. IL‐6, sTNFR‐1, sTNFR‐2 and PAI‐1 remained significantly associated with AKI independent of age, sex, race, clinical interventions and severity of illness markers (hypotension, bilirubin, platelets, degree of hypoxemia and presence of infection) (Darmon et al. 2014). sTNFR‐1 and sTNFR‐2 are soluble tumor necrosis factor receptors that serve as markers of endothelial dysfunction and inflammation, whereas PAI‐1 is a marker of disordered coagulation. These findings support the hypothesis that soluble inflammatory cytokines as well as endothelial dysfunction are important in the pathogenesis of AKI in ARDS. The temporal sequence of these results should be interpreted cautiously: AKI itself can increase cytokines and the pathogenesis of AKI may be underway before a rise in creatinine can be detected.

### 4.3 Organ Support: Impact of Mechanical Ventilation and ECMO

The largest body of evidence regarding lung‐kidney crosstalk after ARDS surrounds the impact of mechanical ventilation on renal function. Mechanical ventilation is frequently required for patients with ARDS. Though mechanical ventilation can be lifesaving, it is not without risk and can itself exacerbate pulmonary damage through ventilator‐induced lung injury (VILI). In clinical studies, mechanical ventilation is independently associated with the risk of developing AKI (Darmon et al. 2014; van den Akker et al. 2013) and increased mortality for patients with AKI (Uchino et al. 2005). Mechanical ventilation may impact renal function through hemodynamic changes affecting renal perfusion, production of inflammatory cytokines, induction of renal tubular apoptosis and neurohormonal mechanisms (Kumar et al. 2024).

Positive pressure mechanical ventilation (PPMV) puts patients at risk for hemodynamic shifts that may impair renal perfusion and promote kidney injury. Hemodynamic changes, particularly renal ischemia due to hypoperfusion, are central to the pathogenesis of AKI in critically ill patients (Juncos et al. 2022). Increasing intrathoracic pressure from PPMV during inspiration increases right atrial pressure, decreases venous return to the right ventricle (RV) and reduces cardiac output (Cortes‐Puentes et al. 2018). PPMV is also associated with an increase in pulmonary vascular resistance which increases RV afterload. Positive end expiratory pressure (PEEP) is used in the management of ARDS to maintain alveolar recruitment, but it also creates increased thoracic pressure throughout the cardiac cycle which can limit venous return during expiration. Furthermore, at high levels PEEP can result in diminished cardiac output due to impairment of left ventricular filling (Jardin et al. 1981). A retrospective analysis of ARDS Network patients found that driving pressures > 15 cm H20 were independently associated with the development of late AKI (two or more days after ARDS diagnosis) (Andrianopoulos et al. 2025). In a rat model of lung injury via intratracheal LPS instillation, a higher PEEP ventilation strategy was associated with increased renal gene expression of NGAL, a tubular injury marker, compared with a lower PEEP approach (Felix et al. 2022).

In humans, injurious mechanical ventilation strategies generate inflammatory cytokines associated with AKI. Lower tidal volume or lung protective ventilation is a cornerstone of treatment for intubated patients with ARDS (Wick et al. 2024). The ARDS Network ARMA trial found that ventilating patients with ARDS using a lower tidal volume strategy decreases mortality compared with conventional ventilation (Brower et al. 2000). Lung protective ventilation is also associated with reduced inflammatory biomarkers that increase the risk of AKI. In a secondary analysis of the ARMA trial, the lower tidal volume group had a greater decrease in IL‐6 and IL‐8 levels from baseline compared with the traditional ventilation group (Parsons et al. 2005). In ARMA, patients with lower tidal volume ventilation had an increased incidence of renal failure‐free days compared with traditional ventilation (Brower et al. 2000). In a small trial of 44 patients with ARDS, Ranieri et al. compared measurements of biomarkers in the plasma and pulmonary aspirates of patients ventilated with a pressure‐limited lung protective strategy vs. conventional approach (Ranieri et al. 1999). Inflammatory biomarkers IL‐6, TNF‐α, IL‐1β were higher in the conventional ventilation group compared with the lung protective group. These studies establish that cytokines associated with AKI are differentially produced in response to mechanical ventilation strategy.

Mechanistic evidence from animals further supports the notion that ventilator strategy can increase the risk of renal damage in ARDS through inflammatory mediators, induction of apoptosis and neurohormonal mechanisms (Kumar et al. 2024). Hepokosi et al. found that markers of endothelial dysfunction—VEGF, VCAM‐1, and Ang‐2—were increased in renal tissue of mice with VILI (Hepokoski et al. 2017). Ang‐2 has been associated with the risk of severe AKI for patients with sepsis (Yu et al. 2021). Imai et al. used a model of acid‐aspiration lung injury in rabbits that were mechanically ventilated with either injurious or noninjurious settings to study the effects on the kidney (Imai et al. 2003). The injurious ventilation group had increased serum creatinine and evidence of renal tubular epithelial cell apoptosis compared with the noninjurious ventilation group. Levels of IL‐8 and MCP‐1 were higher in plasma and pulmonary aspirates from the injurious ventilation group. MCP‐1 has previously been implicated in renal tubular cell apoptosis (Tesch et al. 1999). In an ex vivo model, plasma from the injurious ventilation group induced renal cell apoptosis compared with plasma from the noninjurious group, implicating soluble factors in causing the injury. In contrast, in a canine model of acid‐aspiration lung injury, kidney injury was not detected by serum creatinine or glomerular filtration rate after injurious mechanical ventilation; the lack of AKI was suspected to be due to the similarity of hemodynamic parameters between the injurious and noninjurious ventilation groups (Hoag et al. 2008).

Extracorporeal membrane oxygenation (ECMO) may be initiated in selected cases of severe ARDS where mechanical ventilation cannot adequately address oxygenation and/or ventilation needs (Combes et al. 2025). Broadly, patients with respiratory failure who require ECMO are at increased risk for AKI (Joannidis et al. 2020). It remains undetermined if this phenomenon is due to the increased illness severity of patients requiring ECMO or due to a feature of ECMO itself. ECMO affects fluid balance, hemodynamics, hemolysis, inflammation and endothelial function which all may theoretically affect AKI pathophysiology (Joannidis et al. 2020). In the randomized EOLIA trial of ECMO in ARDS, the ECMO group had significantly more days free of RRT compared with controls (Combes et al. 2018). These results support the hypothesis that worse renal outcomes among ARDS patients requiring ECMO are due to the overall severity of illness prompting ECMO initiation rather than ECMO itself; however, this remains an area of ongoing investigation.

### 4.4 Fluid Management Strategies

Careful fluid management is critical for patients with ARDS, and conservative fluid approaches are recommended (Wick et al. 2024). Theoretically, conservative fluid approaches could unintentionally impair kidney function if hypovolemia is induced. Importantly, in a randomized clinical trial of fluid management for patients with acute lung injury, a conservative fluid strategy did not significantly increase serum creatinine or RRT initiation compared with a liberal fluid strategy (Wiedemann et al. 2006). In a secondary analysis of this trial, AKI was tested as an outcome to determine whether a net positive fluid balance might actually delay the diagnosis of AKI due to falsely low serum creatinine levels from dilution (Liu et al. 2011). Investigators determined if patients in the trial met criteria for AKI diagnosis using two methods: based on raw serum creatinine values or after adjusting the serum creatinine for the underlying balance of fluid intake and output. Before adjusting for fluid balance, the incidence of AKI appeared higher in the conservative fluid group, but after adjusting for fluid balance, AKI incidence was greater in the liberal fluid group (Liu et al. 2011). Adults and children with ARDS who have AKI that is masked by fluid overload have increased mortality comparable with patients who have explicit AKI (Liu et al. 2011; Dixon et al. 2023). Together these results suggest that in patients with ARDS, volume overload may mask new AKI diagnoses and is associated with worse renal outcomes. Conservative fluid management strategies are currently recommended for the management of ARDS, and careful attention to avoiding both renal hypoperfusion and frank volume overload may be beneficial on the risk of AKI (Wick et al. 2024; Ostermann et al. 2025).

## 5 Conclusions

Mechanistic studies using preclinical models have revealed several pathways by which injury in either the kidney or lung can exacerbate injury in the other organ through crosstalk (Figures 1 and 2). Broadly within this crosstalk literature, there are more clinical and epidemiologic studies focused on AKI after ARDS, and there are more mechanistic animal studies examining lung injury after AKI. Advancing the understanding of lung–kidney interorgan communication will require additional mechanistic animal studies of AKI after ARDS, as well as and more clinical and epidemiologic evidence of ARDS following AKI. Challenges in elucidating specific mechanisms of crosstalk in organ injury apply to both preclinical and clinical studies: causal determinants are difficult to isolate in the presence of shared underlying triggers for both lung and kidney injury (e.g., sepsis), and yet, within the clinically defined syndromes of AKI and ARDS there are heterogeneous subpopulations that likely have distinct underlying pathophysiology.

Despite a robust clinical literature on the development of AKI after ARDS and the association of ventilation strategy with AKI, there remains a significant gap in the preclinical literature on specific mechanisms by which lung injury may initiate or exacerbate kidney injury. Pneumonia and sepsis are the most common causes of lung injury in clinical practice and animal models of these conditions are frequently employed. Elucidating causal mechanisms of multiorgan injury with infection or sepsis models is challenging because sepsis is a systemic disease that causes both lung and kidney injury. Is kidney injury after lung injury in the setting of pneumosepsis due to the lung injury or the systemic effects of sepsis? The study of AKI induced lung injury has been propelled forward using primarily non‐infectious models (ischemia and nephrectomy). Though these models may not replicate the full range of pathophysiology contributing to clinical AKI, they are appealing since the temporal sequence of organ injury is clear. Together, studies that use and compare these models have built a compelling story regarding crosstalk from the injured kidney to the lung. Comparisons between models of lung injury, including leveraging non‐infectious models, will help fill the gaps in our understanding of how lung injury impacts the kidney. Comparisons between direct and indirect kidney injury may also be informative as has been done for lung injury (e.g., (Komaru et al. 2025)). Infectious models of sepsis should continue to be used. Careful experiments that simultaneously measure the timing and components of both lung and kidney injury in different types of infectious models may be instructive. Ongoing collaboration between experts in modeling both kidney and lung injury will be essential to further unpacking the onset of multiorgan failure.

From a clinical perspective, studying crosstalk in organ injury is limited by the problem of heterogeneity within these clinical diagnoses. Sepsis, ARDS and AKI are all heterogeneous clinical syndromes that can be subdivided into groups, or phenotypes, with different features and outcomes (Zarbock et al. 2023; Sinha et al. 2023; Bhatraju et al. 2019). The ultimate goal of phenotyping research is to move from identifying phenotypes, groups defined by shared clinical and even biologic characteristics, to identifying true biologic endotypes, defined by shared underlying pathophysiologic mechanisms that could be targeted through precision medicine therapies (Shah et al. 2021; Gordon et al. 2024). The current status of phenotyping for ICU syndromes has been well‐covered by recent reviews (Ostermann et al. 2025; Sinha et al. 2023; Shah et al. 2021; Gordon et al. 2024). It is possible that mechanisms of organ crosstalk after injury are different depending on phenotypes, though there are currently limited studies exploring this question (Famous et al. 2017).

In summary, lung and kidney function are interconnected for patients in the ICU. AKI and ARDS have complex and overlapping epidemiology. Kidney injury can induce pulmonary injury through leukocyte recruitment to the lungs, inflammatory signaling, activation of pattern recognition receptors, formation of neutrophil extracellular traps, osteopontin signaling, metabolic and mitochondrial dysfunction, and impaired ion and water transport. Pulmonary injury can induce kidney injury through inflammatory signaling and is significantly impacted by mechanical ventilation and fluid management approaches.

## Author Contributions

K.M.S., K.D.L. and M.A.M. conducted the literature search and designed the review. K.M.S. wrote the original draft of the manuscript. K.D.L. and M.A.M. critically reviewed and revised the manuscript. All authors approved the final manuscript.

## Conflicts of Interest

The authors declare no conflicts of interest.

## Data Availability

Data sharing not applicable to this article as no datasets were generated or analyzed during the current study.
